# Genistein: A Novel Anthocyanin Synthesis Promoter that Directly Regulates Biosynthetic Genes in Red Cabbage in a Light-Dependent Way

**DOI:** 10.3389/fpls.2016.01804

**Published:** 2016-12-01

**Authors:** Na Zhang, Yan Qi, Hai-Jun Zhang, Xiaoyun Wang, Hongfei Li, Yantong Shi, Yang-Dong Guo

**Affiliations:** ^1^College of Horticulture, China Agricultural UniversityBeijing, China; ^2^Beijing Agriculture Technology Extension StationBeijing, China; ^3^Beijing Vegetable Research Center (BVRC), Beijing Academy of Agriculture and Forestry SciencesBeijing, China

**Keywords:** anthocyanin, antioxidant activity, gene expression, GNT, red cabbage

## Abstract

Genistein (GNT), an isoflavone, is used in the clinical treatment of various health disorders. GNT is found in primary food source plants and some medical plants. However, studies on the functions of GNT in plants are rarely reported. In this study, we demonstrated that GNT plays an important role in promoting anthocyanin accumulation in red cabbage. GNT solutions (10, 20, 30, 40, and 50 mg/L) as foliar fertilizers were applied to red cabbage. Consequently, anthocyanin accumulation in red cabbage increased in a light-dependent manner. GNT solution at 30 mg/L exhibited the optimal effect on anthocyanin accumulation, which was twice that of the control. Quantitative real-time PCR analysis indicated that GNT application upregulated the expression of all structural genes, contributing to anthocyanin biosynthesis under light conditions. Under dark conditions, GNT exerted no significant promotive effect on anthocyanin accumulation; only early biosynthetic genes of anthocyanin biosynthesis responded to GNT. The promotive effect of GNT on anthocyanin biosynthesis is directly attributable to the regulation of structural gene expression. Transcription factors exhibited no response to GNT. The levels of anthocyanin in red cabbage positively correlated with the enzyme activities of antioxidant systems. This finding correlation suggested that the promotive effect of GNT on anthocyanin levels was correlated with improved antioxidant activity in the red cabbage.

## Introduction

Genistein (GNT) is the common name of 5, 7-dihydroxy-3-(4-hydroxyphenyl)-4H-1-benzopyran-4-one. This compound belongs to the group of isoflavones, heterocyclic polyphenols that naturally occur in plants; thus, this compound is also referred to as 4′, 5, 7-trihydroxyisoflavone. GNT is a soy-derived biologically active isoflavone that exerts diverse health-promoting effects. It exhibits numerous biological activities: tyrosine kinase inhibition, chemoprotective activity against cardiovascular disease, and phytoestrogen activity. GNT can impede cancer progression by promoting apoptosis, inducing cell cycle arrest, modulating intracellular signaling pathways, as well as inhibiting angiogenesis and metastasis of neoplastic cells (Wei et al., 1995; Xia and Weng, 2010). A micronutrient with multifaceted effects, GNT should be developed further for its clinical use in the prevention and treatment of various health disorders.

Flavonoids comprise a major class of secondary plant metabolites. Among these metabolites, anthocyanins are the most conspicuous class because of the wide range of colors resulting from their synthesis (Tahara, 2007). Anthocyanins are commonly found in the red, blue, and purple colors of fruits, vegetables, flowers, and other plant tissues (Mateus et al., 2001). As a group of flavonoid compounds, anthocyanins protect plants against various biotic and abiotic stresses, as well as provide flowers and fruits with distinct colors to attract insects and animals for pollination and seed dispersal (Harborne and Williams, 2000). Numerous simple flavonoid compounds also exhibit antioxidant properties and can thus be potentially used as dietary nutraceutics for human health (Winkel-Shirley, 2001). Anthocyanin biosynthesis has been studied extensively (Grotewold, 2006), and its biosynthetic pathway is well described in *Arabidopsis* and other plants, including *Antirrhinum majus* (snapdragon) and *Petunia hybrid* (petunia). Early biosynthetic genes are in the upstream of late biosynthetic genes. Early biosynthetic genes include chalcone synthase (CHS), chalcone isomerase (CHI), flavanone 3-hydroxylase (F3H), and flavonoid 3′-hydroxylase (F3′H), which are common to different flavonoid subpathways (Zhang et al., 2016). Late biosynthetic genes include dihydroflavonol 4-reductase (DFR), leucoanthocyanidin oxygenase (LDOX), and UDP-glucose: flavonoid 3-*O*-glucosyl-transferase (Pelletier et al., 1997). The expression of these structural genes is closely related to anthocyanin levels. Regulatory genes influence the intensity and pattern of anthocyanin biosynthesis by regulating the expression of the structural genes. Many regulatory genes, such as transcription factors R2R3 MYB, basic helix–loop–helix (bHLH), and WD40 proteins, have been cloned from many plants. R2R3 MYB is a transcription factor family carrying the highly conserved R2R3 DNA-binding domain. WD40 is a protein family with WD-repeat sections. They form MBW (MYB-bHLH-WD40) complexes to activate the expression of genes along anthocyanin biosynthesis (Broun, 2005). MYB and bHLH transcription factors contribute differently in activating anthocyanin biosynthetic genes among different plant species (Gonzalez et al., 2008). This variation suggests that unique species-specific regulation of structural genes R2R3 MYB and bHLH transcription factors represent the two major families of anthocyanin regulatory proteins. In *Arabidopsis*, anthocyanin accumulation has been shown to be mediated by 4 MYB proteins and 3 bHLH proteins (Yuan et al., 2009).

Anthocyanin biosynthesis is also influenced by many environmental factors, such as drought (Yuan et al., 2009), temperature (Ubi et al., 2006), hormone (Jeong et al., 2010), or light (Tan, 1980). Red cabbage (*Brassica oleracea* L. var. capitata) grows worldwide as a fresh market vegetable and is a native crop in the Mediterranean region in Europe. Red cabbage is distinct in its high anthocyanin content. It serves as a functional vegetable and is very popular in salad (Charron et al., 2007). To promote anthocyanin accumulation, application of plant growth regulators has been proposed as an economically viable alternative. Many plant growth regulators have been evaluated for regulating anthocyanin biosynthesis in plant tissues. These regulators include gibberellins (Martinez et al., 1996), auxins (Jeong et al., 2010), cytokinins (Kim et al., 2006), ethylene (El-Kereamy et al., 2003), and jasmonate (Ayala-Zavala et al., 2005). Both genetic and physiological approaches have verified that abscisic acid (ABA) positively modulates, whereas gibberellic acid (GA) negatively modulates anthocyanin accumulation on hormone mutants and exogenous applications (Carvalho et al., 2010). In seed germination, ABA and GA were also described as a pair of antagonists (Ho et al., 2003). GA can induce the degradation of DELLA proteins, whereas ABA can cause the stabilization of DELLA proteins (Achard et al., 2006). Meanwhile, a positive role for DELLA proteins in anthocyanin accumulation was demonstrated during phosphorus starvation in *Arabidopsis* (Jiang et al., 2007). DELLA proteins may mediate the antagonism between ABA and GA in anthocyanin biosynthesis. ABA is proven to induce phenylalanine ammonialyase (PAL), a key enzyme for anthocyanin biosynthesis. However, this process does not occur on every species (Guo and Wang, 2009). In addition to these hormones, several chemicals have been found to increase anthocyanin biosynthesis. In the current study, we selected 3 chemicals that naturally exist in plants. 5-Aminolevulinic acid (ALA) is the first compound in the porphyrin synthesis pathway. ALA promotes anthocyanin accumulation in apple (Xie et al., 2013). Guanosine 3′, 5′ -cyclic monophosphate (cGMP) is an important signaling molecule that, as a second messenger in plants, controls various cellular functions. Cyclic GMP regulates the transcriptional activation of the anthocyanin biosynthetic pathway in soybeans (Suita et al., 2009). Melatonin is a chemical that benefits stress tolerance in plants (Zhang et al., 2014, 2015; Arnao and Hernández-Ruiz, 2015; Shi et al., 2016). In our previous study, melatonin treatment increased anthocyanin accumulation in cabbage, *Arabidopsis*, and tomato (Sun et al., 2015, 2016; Zhang et al., 2016).

The effects of GNT in plants remain poorly understood. The mechanism underlying the regulation of anthocyanin accumulation by GNT remains unknown. In the current study, we applied GNT as a foliar fertilizer to evaluate the effect of GNT applications on red cabbage. To determine how GNT improves anthocyanin accumulation, we evaluated the expression of structural and regulatory genes that contribute to anthocyanin synthesis. Light is an essential factor in anthocyanin biosynthesis. Thus, we evaluated the gene expression under light and dark conditions and found that light is an essential factor in GNT-induced anthocyanin accumulation.

## Materials and Methods

### Plant Materials

The following experiments were conducted at China Agricultural University, Beijing (39.9° N, 116.3° E). Cabbage (*Brassica oleracea* var. *capitata* L.) seeds were obtained from Chinese Academy of Agricultural Sciences. After germination, seeds were sown and grown in pots (20 cm in diameter) filled with soil (peat: vermiculite = 2:l) in a growth chamber at 25°C for 10 h during the day and 15°C for 12 h during the night. The light intensity was 600 μmol s^-1^ m^-2^.

### Reagents

All chemicals used in the experiments were of analytical grade. GNT, 5-aminolevulinic acid, and ABA were purchased from Sigma–Aldrich (St. Louis, MO, USA). All other reagents were supplied by Sinopharm Chemical Reagent Beijing Co., Ltd. in China.

### Plant Treatments

For the pre-experiment, solutions of GNT (25 mg/L), 5-aminolevulinic acid (300 mg/L), ABA (600 mg/L), cGMP (80 mg/L), and melatonin (200 mg/L) as foliar fertilizers were applied to 4-week-old red cabbage. The experiment was repeated three times. For the experiments involving GNT, the concentrations of GNT solutions were 10, 20, 30, 40, and 50 mg/L; water was used as the control (with 0.01 % Tween-20). Each treatment consisted of 20 pots, with each pot having 1 plant. All treatments were conducted in triplicate. For the dark treatment, we turned off the light in chamber after the chemical treatment. For the light treatment, the light intensity was 600 μmol s^-1^ m^-2^. The first and the second leaves were collected 5 days post-treatment. All samples were frozen in liquid nitrogen and stored at –80°C for anthocyanin measurement, RNA extraction, and other analyses.

### Analyses of Total Anthocyanin and Total Chlorophyll

Total anthocyanins were measured using a slightly modified differential pH method, a spectrophotometric technique (Rapisarda et al., 2000). Frozen samples (100 mg) were ground into powder in a mortar. Anthocyanin was separately extracted in a pH 1.0 buffer (50 mM KCl +150 mM HCl) and a pH 4.5 buffer (400 mM sodium acetate + 240 mM HCl). The extracts were centrifuged at 12,000 *g* for 15 min at 4°C. Supernates were collected and diluted to measure the absorbance at 510 nm. Total anthocyanin content was calculated using the following equation.

Anthocyanin (mg⋅g^-1^ FW) = (A _pH1.0_ – A_pH4.5_) × 484.8 × 1000/24,825 × dilution factor.

In the formula, 484.8 represents the molecular mass of cyanidin-3-glucoside chloride, and 24,825 is equal to its molar absorptivity (𝜀) at 510 nm. Each sample was analyzed in triplicate, and the results were expressed as the average of the three measurements.

For chlorophyll measurement, we extract chlorophyll by 80% chilled acetone (v/v). Then we used a spectrophotometer to quantify it.

### RNA Extraction and Quantitative Real-Time PCR Analysis

Total RNA was isolated from the samples by using TRIzol Reagent according to manufacturer’s protocol (Invitrogen, Burlington, ON, Canada). The cDNA was reverse-transcribed into cDNA using the reverse transcription system (Takara Biotechnology, Japan). Quantitative RT-PCR was conducted using the Applied Biosystems 7500HT Fast Real-Time PCR System (Applied Biosystems, USA). The reaction volume was 20 μL, containing 2 μL of cDNA, 0.4 μL of each 10 μM forward and reverse primers, and 10 μL of SYBR Premier Ex Taq mix (Takara, Japan). The PCR thermal cycling parameters were 95°C for 10 s, followed by 40 cycles at 95°C for 5 s, 60 °C for 15 s, and 72 °C for 30 s. Melting curve analysis of Quantitative real-time PCR samples revealed only one product for each gene primer reaction, confirming specific amplification. Gene expression was evaluated using the 2^-ΔΔCt^ method. All Qrt-PCR reactions were normalized using the *BoActin* gene (Mittler, 2006). The DNA sequences of PCR primers are listed in **Table 1**.

**Table 1:** Oligonucleotide primers used for qRT-PCR analysis.

Target gene	Primer name	Sequence of primer	Accession	*Arabidopsis* blastN AGI
BoACTIN	BoACTIN-F	CTGTGACAATGGTACCGGAATG	AF044573	AtACTIN2/AT3G18780
	BoACTIN-R	ACAGCCCTGGGAGCATCA		
BoPAL	BoPAL-F	CAGAGCAACACAACCAAGACGTGAA	BH716217	AtPAL1/AT2G37040
	BoPAL-R	TCTCCTCCAAGTGTCGTAGATCGATG		
BoCHS	BoCHS-F	GCGCATGTGCGACAAGTCGAC	EF408921	AtCHS/AT5G13930
	BoCHS-R	CCTGTCGAGCGTCGAGAGAAGGA		
BoCHI	BoCHI-F	TCAAGTTGATTCCGTTACTTTTCCA	EU402417	AtCHI/AT3G55120
	BoCHI-R	ATGACGGTGAAGATCACAAACTTTC		
BoF3′H	BoF3′H-F	TTCCGTACCTTCAGGCGGTTATCAA	BH675335	AtF30H/At5g07990
	BoF3′H-R	CTTTGGGGATATGATAGCCGTTGATC		
BoDFR	BoDFR-F	GCTCTCTCCTATCACTCGTAACGA	AY228487	AtDFR/AT5G42800
	BoDFR-R	GTCGCATCGTGAGAGGAACAAA		
BoLDOX	BoLDOX-F	GTGGACAGCTTGAGTGGGAAGATTAC	AY228485	AtLDOX/AT4G22880
	BoLDOX-R	GTACTCACTCGTAGCTTCAATGTAATCAG		
BoGST	BoGST-F	CTTGTAGCCATTTGGTCAA	BH738469	AtGSTF12/AT5G17220
	BoGST-R	GAGACTTGCCCAAAAGGTTCGT		
BoTT2	BoTT2-F	AAACCAAGCTGGTCTCAAGAGGTGTG	DQ778648	AtTT2/At5g35550
	BoTT2-R	AACGACCATCTGTTTCCAAGGAGATTAT		
BoTT8	BoTT8-F	CCAATAGTTTAGATACACACATGGACATG	BH450920	AtTT8/At4g09820
	BoTT8-R	TCTTTGACATTCTCAACTCTCCACGATAT		
BoTTG1	BoTTG1-F	AGTTGCAGTGGTCGGCTTCTC	BH653524	AtTTG1/AT5G24520
	BoTTG1-R	AATACGAACCTCAAACTCTAAGGAGCT		
BoEGL3	BoEGL3-F	AACTGTCAATTGCAAGCATAAAGGGACA	EX078387	AtEGL3/At1g63650
	BoEGL3-R	TGTTTGAATCACTGAGTTCATAAGATTGGA		
BoMYB12	BoMYB12-F	TGGAACTCTCATCTCCGCCGTAA	BH539285	AtMYB12/At2g47460
	BoMYB12-R	CGGCGGTGCAGACGTTCTCTT		
BoMYB2	BoMYB2-F	GGAAACAGGTGGTCTTTAATTGCT	N/A	AtMYB114/At1g66380
	BoMYB2-R	AGCTCAAATTTATCATCATCTTTGTTACATGTGATTA		
BoMYB4	BoMYB4-F	GGAAACAGGTGGTCTTTAATTGCT	N/A	AtPAP1/At1g56650
	BoMYB4-R	ATCCAAGGCATAGGGGAACAAAT		

### Evaluation of Lipid Peroxidation

The levels of lipid peroxidation in the leaves were evaluated by protocol we described before (Zhang et al., 2013). The level of lipid peroxidation was expressed as concentrations of malondialdehyde (MDA).

### Antioxidant Enzyme Extraction and Assay

Frozen samples (1 g FW) were homogenized with 0.2 g hydrated PVP (insoluble polyvinylpyrrolidone) in 10 mL of 50 mM phosphate buffer (pH 7.8) supplemented with 2 mM dithiothreitol and 0.1 mM ethylenediaminetetraacetic acid (EDTA) and then centrifuged at 16,000 *g* for 15 min. The resulting supernatant was used for enzyme assays. All steps of the extraction procedure were carried out on ice to make sure the temperature is low.

Superoxide dismutase (SOD; EC 1.15.1.1) activity was measured according to Giannopolittis and Ries (Giannopolitis and Ries, 1977), with certain modifications. You can find the detailed protocol in the paper we pubilished in 2013 (Zhang et al., 2013).

Catalase (CAT; EC 1.11.1.6) activity was measured at 25°C according to Kato and Shimizu (Kato and Shimizu, 1987). The 3 mL reaction mixture contained 3.125 mM H_2_O_2_ in 50 mM phosphate buffer (pH 7.8) and 0.2 mL of enzyme extract. CAT activity was estimated by the absorbance decrease at 240 nm of 0.1 unit/min and was expressed as units g^-1^ min^-1^.

Peroxidase (POD; EC 1.11.1.7) activity was measured at 25°C according to Scebba et al. (2001). The method is based on monitoring the H_2_O_2_ decomposition rate by POD, using guaiacol as a hydrogen donor. The reaction was initiated by adding 50 μL of enzyme extract to 1,950 μL phosphate buffer (65 mM, pH 5.5) containing 11 mM H_2_O_2_ and 2.25 mM guaiacol. The rate of color development was determined by recording the absorbance of the reaction solution at 470 nm per 0.1 s. One unit of POD activity was defined as an absorbance change of 0.01 units/s; activity was expressed as units g^-1^ s^-1^.

### Statistical Analysis

All data were subjected to one-way ANOVA with Duncan’s test or *t*-test in SPSS 20.0.

## Results

### Effects of Five Different Chemicals on Anthocyanin Accumulation in Red Cabbage

**FIGURE 1:**
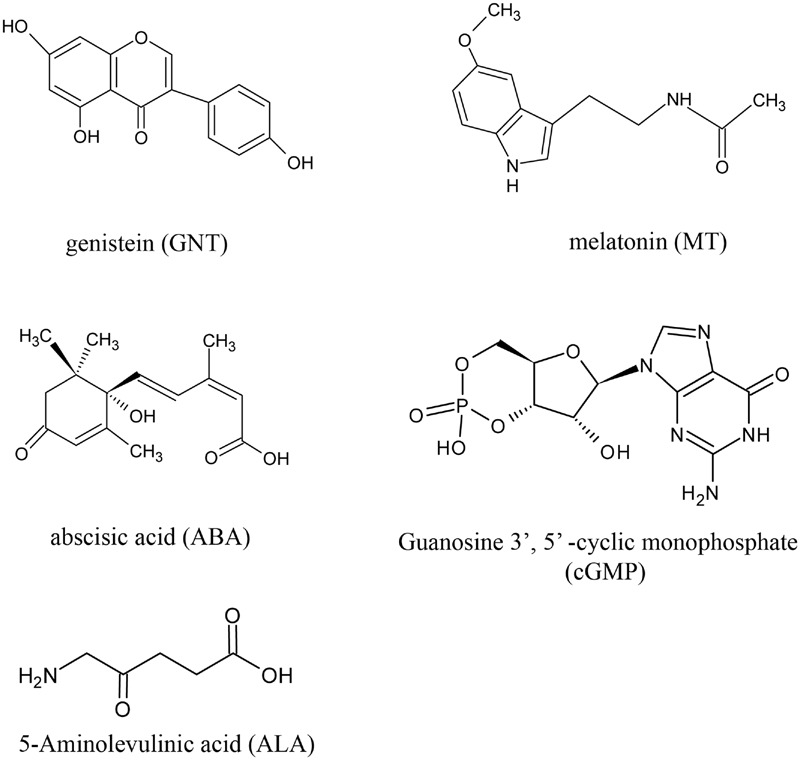
**The structure of the chemicals applied to red cabbage**.

**FIGURE 2:**
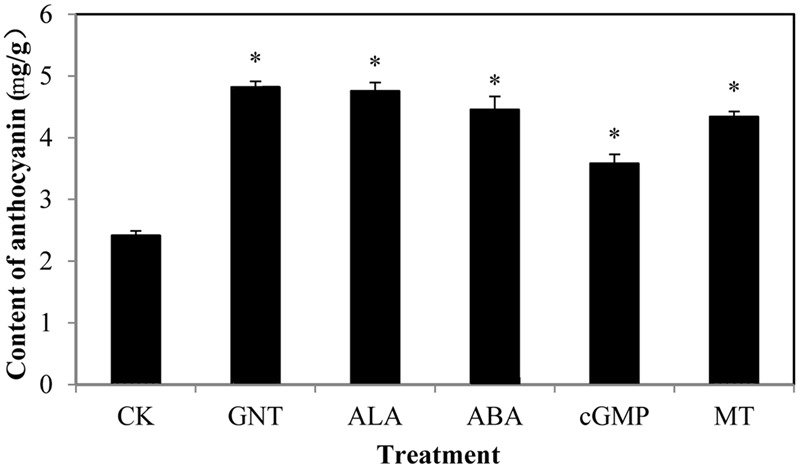
**The content of anthocyanin in red cabbage under four treatments: CK (control), GNT (genistein), ALA (5-Aminolevulinic acid), ABA (abscisic acid), cGMP (Guanosine 3′, 5′-cyclic monophosphate) and MT (melatonin).** The data are *mean* ±*SD* (*n* = 3) and the symbol ^∗^represents significant difference with CK and treatments at *P* = 0.05 level.

We evaluated the anthocyanin levels of red cabbage under five different chemical treatments. **Figure 1** shows the structure of the chemicals used in this study, with GNT as the star chemical. ALA is an intermediate along the chlorophyll biosynthetic chain, which is used to promote the skin color in apple fruits. ABA is an important phytohormone related to stress response, but it is widely reported as an improvement of anthocyanin biosynthesis. Guanosine 3′, 5′-cyclic monophosphate (cGMP), a second messenger, also improves anthocyanin accumulation. Meanwhile, the indole melatonin can accelerate anthocyanin biosynthesis. We gathered these chemicals to evaluate their effects on anthocyanin accumulation. All anthocyanin levels were higher in these treatments than in the controls (**Figure 2**). GNT, ALA, ABA, and MT exerted similar effects on the anthocyanin levels, which were higher compared with cGMP. In our previous study, we evaluated the effect of melatonin on anthocyanin accumulation (Zhang et al., 2016). In the present study, we selected GNT as a subject to evaluate its effect on anthocyanin accumulation.

### Improvement of Anthocyanin Accumulation with Different GNT Concentrations in Red Cabbage

**FIGURE 3:**
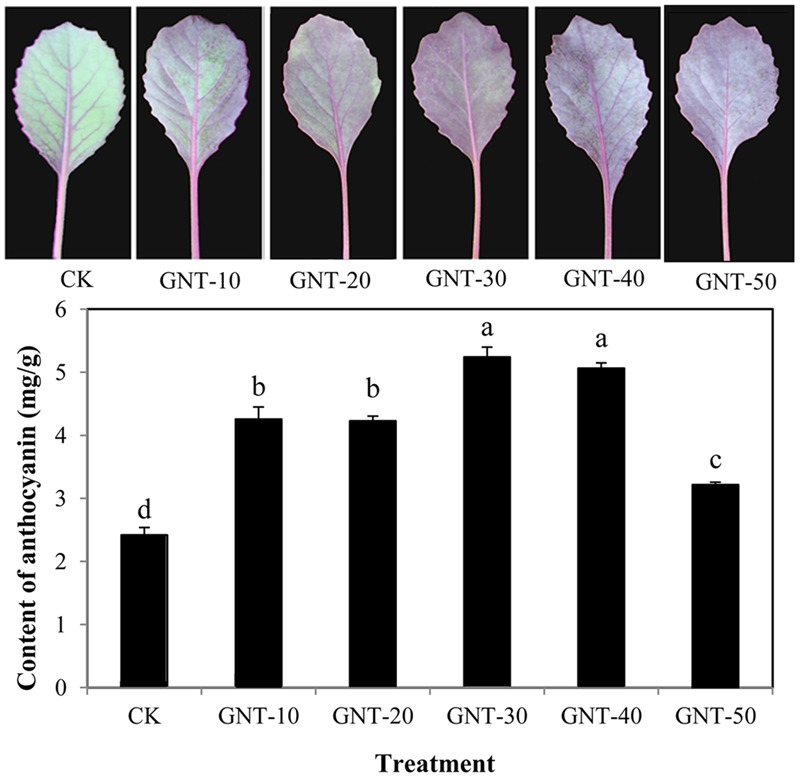
**The content of anthocyanin under different concentrations of genistein: GNT-10 (10 mg/L genistein), GNT-20 (20 mg/L genistein), GNT-30 (30 mg/L genistein), GNT-40 (40 mg/L genistein), GNT-50 (50 mg/L genistein).** The data are *mean* ± *SD* (*n* = 3) and the different characters represent significant difference among treatments at *P* = 0.05 level.

To evaluate the effect of GNT on anthocyanin accumulation in red cabbage, GNT with gradient concentrations were applied to cabbage seedlings. GNT treatments under five different concentrations significantly regulated anthocyanin accumulation in red cabbage (**Figure 3**). GNT with lower concentrations showed higher anthocyanin accumulation levels, with 30 mg/L being the optimal concentration of GNT treatment. The anthocyanin level was 2.4-fold higher under the optimal concentration than in the control, with 5.24 mg/g FW. The highest concentration used in this experiment was 50 mg/L, which also improved anthocyanin accumulation; however, the increase was not as much as that obtained at optimal concentration. These results suggested that GNT could promote anthocyanin accumulation in red cabbage, and the promotive effect was concentration-dependent.

### Light-Dependent Promotive Effect of GNT on Anthocyanin Accumulation

**FIGURE 4:**
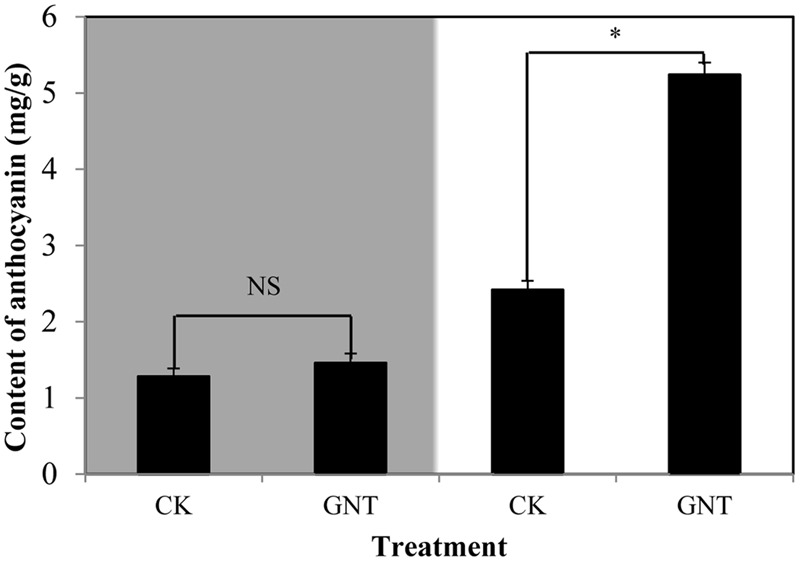
**Anthocyanin levels of red cabbage under GNT treatment in dark and light conditions.** The colored section is dark condition and bright section is light condition. The data are *mean* ±*SD* (*n* = 3) and the symbol ^∗^ represents significant difference with CK and treatments at *P* = 0.05 level while NS means not significant.

To determine whether the promotive effect of GNT on anthocyanin accumulation is light-dependent, we considered light conditions in our experiments. The GNT solution used was the optimal concentration (30 mg/L) obtained, as shown in **Figure 3**. Under dark conditions, anthocyanin levels were similar in the GNT-treated and control groups (**Figure 4**). No significant difference was observed after GNT treatment. While anthocyanin accumulation levels were significantly increased under light conditions in both the GNT-treated groups and the control group. Under light conditions, GNT treatment significantly improved the anthocyanin level to twice that of the control (**Figure 4**). This result indicated that the promotive effect of GNT on anthocyanin accumulation is light-dependent. We also evaluated the chlorophyll content in GNT treatments under dark and light conditions. A difference in chlorophyll content was observed between light and dark conditions but not between the GNT-treated group and the control group (**Table 2**). This finding indicated that GNT treatment exerted no effect on chlorophyll synthesis.

**Table 2:** Chlorophyll content of GNT treatments under dark and light conditions: Chla (chlorophyll a), Chlb (chlorophyll b).

Treatments	Chl a/(mg/g^-1^)	Chl b/(mg/g^-1^)	carotenoid(mg/g^-1^)
CK	0.971 ± 0.027a	0.386 ± 0.006a	0.333 ± 0.013a
GNT	0.901 ± 0.043a	0.343 ± 0.014b	0.312 ± 0.013a
CK	0.725 ± 0.059b	0.300 ± 0.019c	0.271 ± 0.021b
GNT	0.692 ± 0.012b	0.285 ± 0.010c	0.267 ± 0.007b

*Different letters in the same column indicate a significant difference in particular series at *P* = 0.05 according to ANOVA and Duncan’s multiple range tests.*

### Positive Effect of GNT Treatment on the Expression of Genes Along the Anthocyanin Biosynthetic Pathway

**FIGURE 5:**
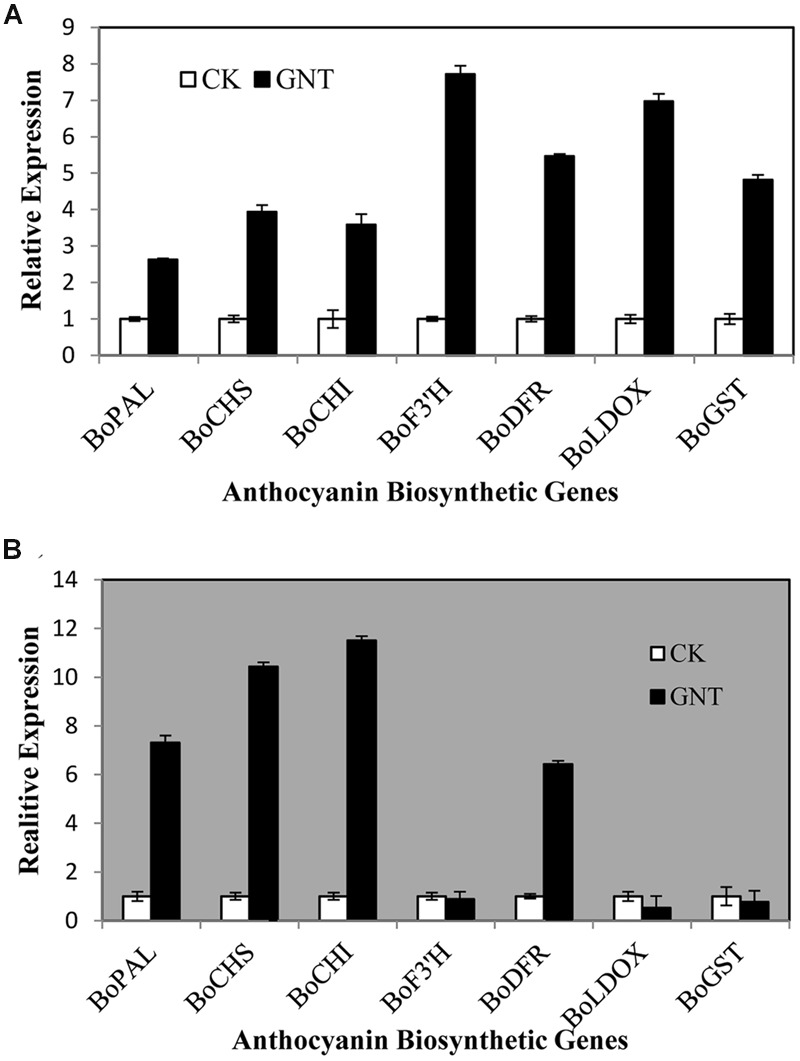
**Expression levels of genes along anthocyanin biosynthesis under genistein treatments. (A)** is under light conditions and **(B)** is under dark conditions. The data are *mean* ±*SD* (*n* = 3), Vertical bars represent ±*SD*.

To investigate whether the GNT induced anthocyanin accumulation is due to the upregulated gene expression, we measured the expression levels of transcripts that encode 7 anthocyanin biosynthetic genes (*BoPAL, BoCHS, BoCHI, BoF3′H, BoDFR, BoLDOX*, and *BoGST*) by Quantitative real-time PCR. The expression patterns of the seven biosynthetic genes under dark and light conditions in red cabbage are presented in **Figure 5**. Under light conditions, the 7 genes showed upregulated expression with GNT treatment. The genes *BoF3′H, BoDFR*, and *BoLDOX*—the key enzymes in the last step of anthocyanin biosynthesis—showed higher upregulation with GNT treatment (**Figure 5A**). Meanwhile, under dark conditions, several genes also exhibited upregulated expression after GNT treatment. These genes included *BoPAL, BoCHS*, and *BoCHI*, which participate in the early steps of anthocyanin biosynthesis (**Figure 5B**). Under dark conditions, genes of the late-step enzymes, which play a more direct role in converting the intermediate products to anthocyanin, showed no significant change in expression with GNT treatment (**Figure 5B**). However, *BoDFR* is an exception.

**FIGURE 6:**
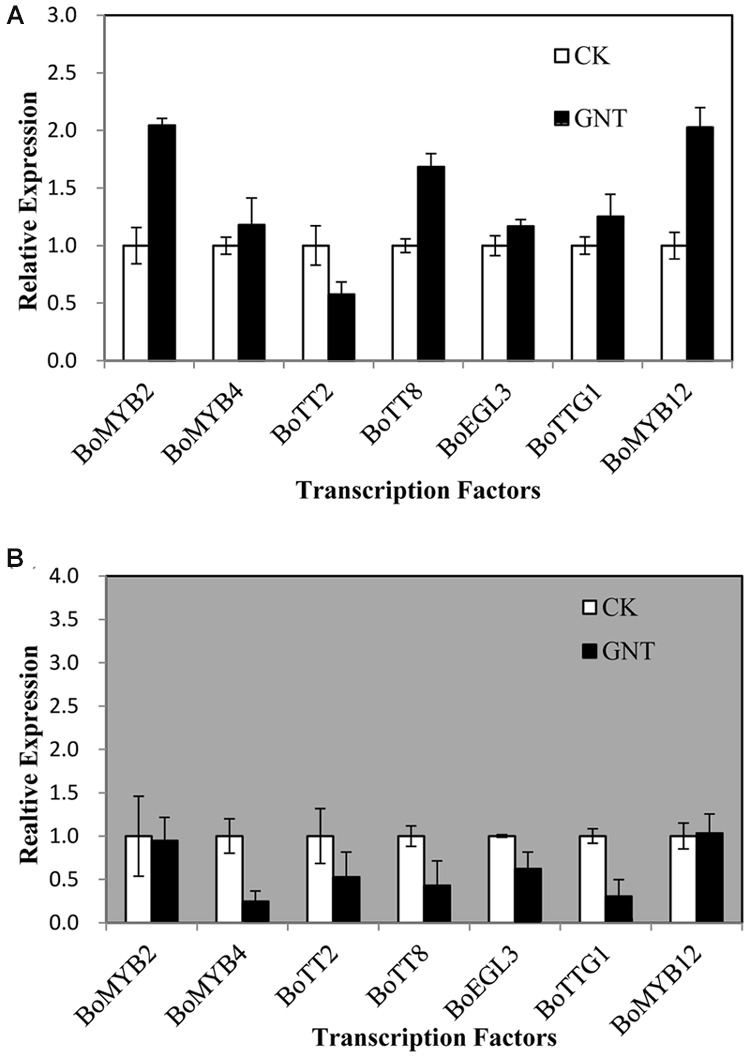
**Expression levels of transcription factors regulating anthocyanin biosynthesis under genistein treatments. (A)** is under light conditions and **(B)** is under dark conditions. The data are *mean* ±*SD* (*n* = 3), Vertical bars represent ±*SD*.

We aimed to investigate whether transcripts corresponding to the anthocyanin regulatory genes accumulated in response to GNT both under dark and light conditions. We also intended to examine possible correlations between their expression patterns and those of the structural genes. We evaluated several transcription factors that were known to regulate the structural genes in the anthocyanin biosynthetic pathway, including *BoMYB2, BoMYB4, BoTT2, BoTT8, BoEGL3, BoTTG1*, and *BoMYB12*. Anthocyanin biosynthetic genes are regulated by the interaction of the transcription factors MYB, bHLH, and WD40. The bHLH transcription factors participate in the regulation of anthocyanin biosynthesis, including TT8 and EGL3. TTG1 is a WD40 protein that correlates with anthocyanin biosynthesis. The remaining transcription factors that were evaluated were MYBs. All transcription factors showed low expression levels with GNT treatment under dark and light conditions (**Figure 6**). The largest upregulation in expression, which was only onefold higher than that of the control, was observed under light conditions in *BoMYB2* and *BoMYB12* (**Figure 6A**). The transcription factor *BoTT2* exhibited a slight downregulation. Under dark conditions, the transcription factors showed no change or downregulation with GNT treatment (**Figure 6B**). These results indicated that GNT may directly regulate the expression of structural genes in anthocyanin biosynthesis instead of by transcription factors.

### Effects of GNT Treatment on the Antioxidant System in Red Cabbage

**FIGURE 7:**
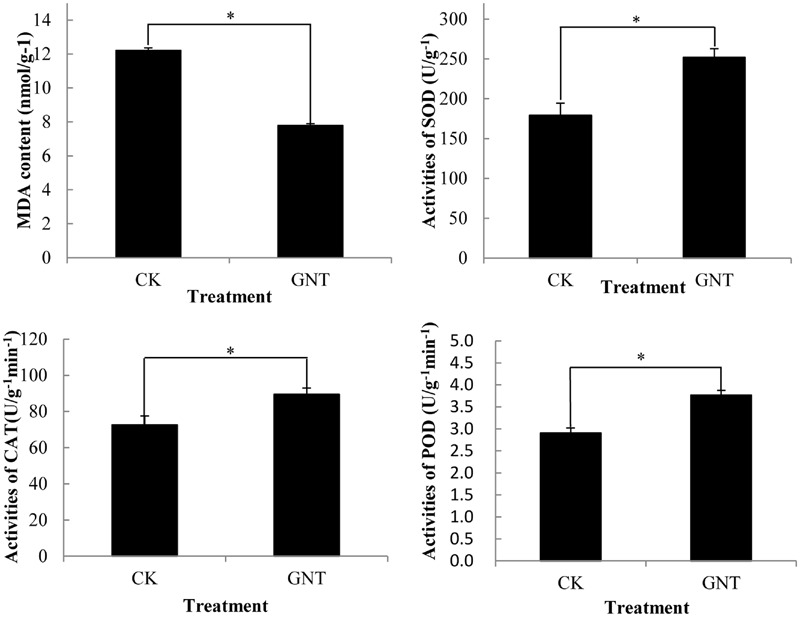
**Genistein treated cabbage showed lower MDA levels and higher antioxidant system enzyme activites.** The data are *mean* ±*SD* (*n* = 3), Vertical bars represent ± SD. The symbol ^∗^ represents significant difference with CK treatment at *P* = 0.05 level.

GNT treatment not only promoted anthocyanin accumulation but also improved antioxidant activity in red cabbage. Reactive oxygen species (ROS) cause lipid peroxidation, which is presented by MDA content. ROS can be used as an efficient indicator of the integrity of cell membranes in plants subjected to ROS stress. In the present study, the GNT-treated groups showed lower MDA content compared with the control group (**Figure 7**). This difference indicated that GNT treatment helped maintain the integrity of cell membranes in red cabbage seedlings. GNT treatment also increased the activities of classical antioxidant enzymes, such as SOD, POD, and CAT. The activities of these enzymes were significantly upregulated by GNT treatment (**Figure 7**). The activities of these enzymes were highly collaborated to anthocyanin levels with GNT treatment. Anthocyanins were reported to possess antioxidant activity and upregulated antioxidant activity may be attributable to upregulated anthocyanin accumulation (Zhu, 2002).

## Discussion

In the current study, GNT exerted a promotive effect on anthocyanin accumulation in red cabbage, similar to the previously reported chemicals, including ALA, ABA, cGMP, and MT (**Figure 2**). No studies have reported on the effect exerted by GNT; thus, we evaluated the effect of GNT on anthocyanin accumulation in detail. We found that 30 mgL^-1^ GNT induced the highest anthocyanin level, which is 2.4-fold that of the CK group (**Figure 3**). Furthermore, the anthocyanin promotive effect is light-dependent. Under dark conditions, no significant change in anthocyanin level was observed (**Figure 4**).

**FIGURE 8:**
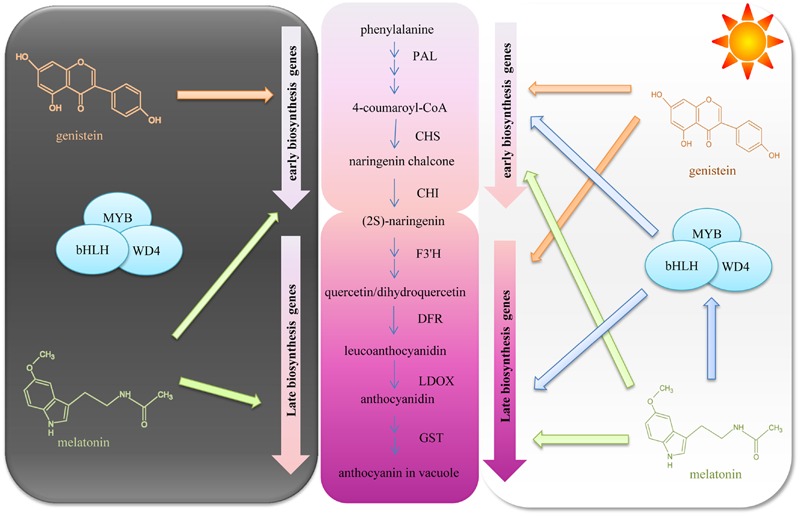
**The mechanisms of genistein regulating anthocyanin biosynthesis in dark and light conditions**.

Anthocyanin biosynthesis is regulated primarily at the transcriptional level. To explore the mechanisms of upregulated anthocyanin accumulation by GNT, we evaluated the expression of several genes and transcription factors in the anthocyanin biosynthetic pathway. **Figure 8** presents the mechanisms identified. The middle section of the graph is the anthocyanin biosynthetic pathway. PAL is the first committed enzyme in the anthocyanin biosynthetic pathway, which leads to the production of numerous flavonoids (Springob et al., 2003). This enzyme catalyzes the formation of *trans*-cinnamic acid from phenylalanine. CHS catalyzes the formation of a triketide intermediate from 4-coumaroyl-CoA and three molecules of malonyl-CoA; subsequently, spontaneous cyclization of triketide intermediate results in the formation of naringenin chalcone. CHI catalyzes the stereospecific cyclization of naringenin chalcone to (2S)-naringenin (Zhang et al., 2016). These genes participate in the early steps of anthocyanin biosynthesis. The products of these steps are also intermediate products of other products. We found that these genes can be regulated by GNT under both dark and light conditions, as shown in **Figure 5**. The compound synthetic pathways are complex, resembling a network. The product of every step is also an intermediate of other compounds. No significant change in anthocyanin levels were observed with GNT treatment under dark conditions (**Figure 4**), maybe some other products share the early steps were upregulated. GNT directly regulated the early biosynthetic genes under dark and light conditions without the participation of transcription factors (**Figure 8**). This process slightly differed from the mechanism of melatonin. Melatonin directly regulates the expression of early genes under both dark and light conditions. However, in the presence of light, melatonin can regulate the genes both directly and with transcription factors (Zhang et al., 2016). F3′H is a cytochrome P450 monooxygenase committed in the hydroxylation of the 3′-position of the B-ring of flavonoid (Holton and Cornish, 1995). This enzyme can accept either dihydrokaempferol or kaempferol as a substrate and convert them to dihydroquercetin and quercetin, respectively. DFR catalyzes a reduction reaction of dihydroflavonol to leucoanthocyanidin. The enzyme LDOX catalyzes the formation of anthocyanidin from leucoanthocyanidin with 2-oxoglutarate and oxygen as co-substrates. GST protein is the flavonoid carrier, forming conjugates with anthocyanins, thereby preventing them from oxidation. It participates in the transport of anthocyanins from the cytosol to the vacuole (Zhang et al., 2016). These genes are late biosynthetic genes in the anthocyanin pathway. Under light conditions, GNT upregulation of these gene expression levels resulted in high anthocyanin biosynthesis (**Figures 4** and **5A**). However, under dark conditions, these late genes exhibited no response to GNT and produced no increase in anthocyanin accumulation (**Figures 4** and **5B**).

In all higher plants studied to date, the anthocyanin pigment pathway is regulated by a suite of transcription factors that include MYB, bHLH, and WD-repeat proteins (Broun, 2005; Gonzalez et al., 2008; Xu et al., 2015). Many signaling molecules affect anthocyanin synthesis by activating the transcription factors. ALA was reported to upregulate these transcription factors (Xie et al., 2013). ABA, jasmonate, or cytokinins induced anthocyanin biosynthesis; GA, ethylene, or brassinosteroids repressed anthocyanin accumulation, which were related to the activation and repression of these transcription factors (Loreti et al., 2008; Carvalho et al., 2010; Jeong et al., 2010). The results of our study indicated that the transcription showed no response to GNT treatment. GNT upregulated the anthocyanin biosynthetic genes. This effect suggested that GNT can be a potential downstream factor along the anthocyanin biosynthetic pathway.

Light is one of the most important environmental factors regulating plant development and gene expression. Light exposure can increase the concentration of anthocyanins by activating many genes along the biosynthetic pathway. Under dark conditions, GNT could only regulate the early genes of anthocyanin biosynthesis (**Figure 8**); under light conditions, all genes along the biosynthetic pathway were activated and exerted a higher expression with GNT treatment. Melatonin regulated the expression levels of both early and late genes under dark conditions but only mildly. Under light conditions, the expression of these genes sharply increased with melatonin treatment (Zhang et al., 2016). Not only the structural genes but the transcription factors as well strongly responded to melatonin under light conditions.

## Conclusion

In this study, we found a novel anthocyanin biosynthesis promoter: GNT. It is a plant-derived molecule that plays a role in maintaining health. We also found that GNT affects anthocyanin biosynthesis in a light-dependent manner. Anthocyanin biosynthetic genes showed an upregulated expression under light conditions. Under dark conditions, only the early biosynthetic genes responded to GNT. GNT directly regulated the structural genes—i.e., without the participation of transcription factors—unlike other signaling molecules. Anthocyanin is shown to be an effective antioxidant. Our study found that GNT treatment also improved the antioxidant activity of red cabbage.

## Author Contributions

NZ, YQ, and Y-DG designed research; NZ, YQ, H-JZ, XW, HL, and YS performed the experiments; NZ, YQ, H-JZ analyzed the data; NZ and YQ wrote the manuscript. NZ and Y-DG revised the manuscript.

## Conflict of Interest Statement

The authors declare that the research was conducted in the absence of any commercial or financial relationships that could be construed as a potential conflict of interest.
